# Diversity of Multidrug Efflux Genes and Phenotypic Evaluation of the *In vitro* Resistance Dynamics of Clinical *Staphylococcus Aureus* Isolates Using Methicillin; a Model β-lactam

**DOI:** 10.2174/1874285801711010132

**Published:** 2017-06-30

**Authors:** John F. Antiabong, Marleen M. Kock, Nontombi M. Mbelle, Marthie M. Ehlers

**Affiliations:** Department of Medical Microbiology, University of Pretoria Medical Microbiology, Pretoria, South Africa

**Keywords:** Methicillin-resistant *Staphylococcus aureus* (MRSA), Multi-drug efflux (MDE) genes, Quantitative analysis, antibiotic adjuvants, β lactam antibiotic

## Abstract

**Objectives:**

Methicillin-resistant *Staphylococcus aureus* (MRSA) across the world often leave clinicians with little or no choice of treatment options. The multi-drug efflux (MDE) genes are bacterial survival mechanisms responsible for the pumping out of antibiotics and other biocides from the cytoplasm. Whilst effort is being made in the development of antibiotic adjuvants such as efflux pumps inhibitors, information is needed on the diversity of these MDEs in the circulating *S. aureus* and on the growth dynamics of the clinical isolates in response to antibiotics is not regularly examined.

**Methods:**

Here, we evaluated the diversity of MDEs in cinical *S. aureus* recovered in a tertiary academic hospital, Pretoria, South African hospital using PCR and also employed visual minimum inhibitory concentration and quantitative analysis of spectrophometric measurements of bacterial growth in the presence of a model β lactam antibiotic (methicillin), to phenotypically elucidate the resistance pattern of these isolates in response to methicillin.

**Results:**

Three major distribution patterns of MDEs were observed in the clinical isolates evaluated. Moreover, *nor*A, *nor* B and *tet*38 were present in 98.9% of the isolates while other MDE were present in different proportions ranging from 40 to 98.6% of the isolates. In addition, *S. aureus* isolates, be it of MRSA or MSSA genotype did not habour the same set of MDEs despite being recovered from the same hospital setting. Finally, we showed that MSSA displayed phenotypic resistance to methicilllin despite the non-detection of the *mec*A resistance gene.

**Conclusions:**

Our data suggest that the growth of *S. aureus* may be enhanced by β lactams (methicillin) and that MSSA may also display resistance to methicillin and perhaps other β lactam antibiotics. The high prevalence of MDEs suggestive of resistance to a broad spectrum of biocides and fluoroquinolones are particularly disturbing.

## INTRODUCTION

MRSA is a Gram positive bacterium with a circular chromosome of 2.2-2.8 Mbp and varieties of extra-chromosomal elements that include plasmids, phages and other mobile elements [1]. The genome of *S. aureus* encodes chromosomal and extra-chromosomal virulence determinants, antibiotic resistance genes and multi-drug efflux genes (MDE) which play significant roles in the pathogenesis of *S. aureus*-associated infections and poor antibiotic therapeutic outcome [2]; thereby increasing patient suffering and sustained epidemics.

The MDE genes are bacterial survival mechanisms responsible for pumping out antibiotics and other biocides from the cytoplasm [3]. These efflux pumps can either be chromosomal or plasmid encoded and are able to extrude specific and/or different classes of antimicrobial compounds including biocides [2]. The MDEs are grouped into five families based on their structure and the kinetics of their activities: (i) the major facilitator superfamily (MFS), (ii) the small multi-drug resistance (SMR) family, (iii) the multi-drug and toxic compound extrusion (MATE) family, (iv) the resistance-nodulation-cell division (RND) superfamily and (v) the adenosine-triphosphate (ATP)-binding cassette (ABC) superfamily. Several MDEs have been described in *S. aureus* Table (**S1**) that contribute to the antimicrobial resistance mechanisms in this pathogen [2].

Previous studies have reported the prevalence and diversity of clinical MRSA isolate in Pretoria, South Africa [4, 5]. However, there are no reports on the distribution patterns of the multidrug efflux genes of the clinical isolates in South Africa. In addition, studies on antimicrobial profiling of *S. aureus* isolates often report the minimum inhibitor concentrations (MIC) of the tested antimicrobial and neglect the evaluation of the growth dynamics of the isolates; an assessment that could provide insight into the growth characteristics of the pathogen in response to antibiotics. This report reveals that all *S. aureus* isolates, be it of MRSA or MSSA genotype, do not contain the same set of MDEs despite being isolated from the same hospital setting and that there are at least four groups of specific growth-response pattern showing resistance to the model I lactam antibiotic, methicillin.

## MATERIALS AND METHOD

### Experimental Design Rationale

The clinical use of methicillin as antibiotics has been halted in many countries due to the development of resistance by microbes to this antimicrobial agent. However, this phenomenon makes it a useful tool in unravelling the resistance mechanisms to β lactam antibiotics for the following reasons: (a) Methicillin forms the chemical backbone for modern β lactam antibiotics. (b) Most clinical *S. aureus* isolates are likely to develop resistance to this chemical agent due to the presence of *mec*A gene in MRSA or due to exposure to modern β lactam antibiotics sharing similar chemical structure (c) Methicillin is insensitive to the *S. aureus* encoded beta lactamase [6] thus, making it possible to rule out the effect of antibiotic degradation. The MDEs investigated in this study are limited to those reported in Costa *et al.* [2] and covers the main classes of MDEs so far identified in *S. aureus* Table (**1**).

### Specimen

Ninety-seven previously characterized Staphyloccocal isolates were obtained from the Diagnostic Laboratory, Department of Medical Microbiology, University of Pretoria Tshwane Academic Division, National Health Laboratory Service, South Africa. These consisted of 81 MRSA and 16 MSSA isolates. The specimen were collected from Tshwane Academic Hospital in Pretoria, South Africa with Research Ethics Approval (protocol number 394/2014) from the Faculty of Health Sciences, University of Pretoria.

### Total Bacterial DNA Purification

Total bacterial DNA purification. Each isolate was plated out on blood agar to ensure the purity and a single colony inoculated in Brain heart infusion (BHI) broth (Oxoid, England) and incubated (Scientific Incubator, Vacutec, South Africa) at 37˚C for 24 h.

The genomic DNA from individual isolates was extracted using the ZR Fungal/Bacterial DNA Miniprep (Zymo Research Corporation, USA) according to the manufacturer’s instruction and the specific genes encoding multi-drug efflux genes were detected by multiplex-PCR using target-specific primers (Table **1**).

### Multiplex-PCR Assays

The amplification of the target genes was performed in a 25 µl reaction mix (Table **S2**) using a cycliplex PCR strategy [7] which involves a single optimized-PCR condition that allows multiplexing of different gene target-primer sets in different PCR tubes and run at once thereby saving time (Table S2). The identity of each isolates as MRSA or MSSA was reconfirmed by PCR detection of the *S. aureus mec*A genes as previously described [4, 8]. To assess the general PCR performance, the eubacterial 16S rDNA V3 region was also targeted in each batch of PCR run as described previously [7].

All PCR amplicons were fractionated in 2.5% agarose gel (NuSieve ™ GTG ™ agarose, Whitehead scientific, South Africa) and visualized under UV light for digital documentation. A 100 bp DNA ladder (Thermo Scientific, South Africa) was included in the electrophoresis run for estimation of the amplicon size. The distribution of the MDEs in each isolate was recorded.

### Determination of Minimum Inhibitory Concentration (MIC), Susceptibility Concentration (SC) and Bacteria Growth Dynamics

A total of 24 isolates consisting of a minimum of two isolates from a cluster of MRSA or MSSA as determined by UPGMA (Unweighted Pair Group Method with Arithmetic Mean) clustering of the MDEs distribution in each isolate Fig. (**S1**), were used for this assay. Single outliers that did not cluster with any other isolate were also included. The methicillin (Sigma Aldrich; South Africa) SCs for the 24 clinical MRSA and MSSA isolates were determined using the micro-dilution method as described in the Clinical and Laboratory Standards Institute (9). Briefly, 5 X 10^4^ CFU/ml of each isolate was incubated in Tryptone soya broth (Oxoid, South Africa) in the presence of 0.5, 1, 2, 4, 8 and 16 µg/ml methicillin for 16 hr at 37 ˚C. The visual SCs were defined as susceptible (no visual growth observed); resistant (micro-well completely covered with bacteria; and intermediate resistance (about 1-4 dot-like growth observed in the micro-well). Two independent tests were performed to assess reproducibility. The MIC was taken as the minimum concentration in which no visual bacterial growth was observed. The growth of the 24 *S. aureus* isolates was also determined by quantitative analysis of spectrophotometric measurements of the optical density (absorption) at 600_nm_ after the 16 h incubation period. The *S. aureus* MU50 ATCC 700699 was used as control in the spectrophotometric measurements.

### Statistics

The distribution pattern of MDEs in each MRSA/MSSA and the growth dynamics of representative isolates were evaluated using a UPGMA (Unweighted Pair Group Method with Arithmetic Mean) dendrogram constructed by hierarchical clustering with a Jaccard Tanimoto co-efficient and a distance value estimated using the formula d = (1-r) X 100. The results are presented in a table format for clarity.

A correlation statistics of the co-existence of MDEs in the MRSA and MSSA isolates was determined by Pearson’s correlation with *p* ≥ 0.05 and the result rendered as a heatmap matrix with the aid of Plotly, an open source program (https://plot.ly/).

## RESULTS

### Diversity and Distribution Pattern of MDEs in Clinical *S. aureus* Isolates

In other to evaluate the distribution pattern of the MDEs, all the isolates were reconfirmed as MRSA or MSSA and the PCR strategy used for the MDEs used proved to be robust and showed distinct and clear bands on the electrophoretic gel (Fig. **S2**). The distribution pattern of the detected MDEs in the clinical MRSA and MSSA isolates clustered into three major groups (Fig. S1 and Table S3). All the 11 MDE genes assessed in this study were detected in 35.05% (34/97) of the total isolates; out of which, 8.82% (3/34) were MSSA. The *smr* and *qac*A/B genes were not detected in 31.96% (31/97) of the all isolates, which, however; harboured nine other MDEs. The MSSA represented 16.1% (5/31) of this cluster. Moreover, MDEs including *nor*C, *sdr*M and *qac*A/B were not detected in 12.37% (12/97) of the isolates, which hitherto had 8 other MDEs. This cluster included 50%(6/12) MSSA isolates. Other clusters/outliers included isolates numbers ranging from 1-4 and made up to 21.2% of the total *S. aureus* isolates. We did not observe any correlation between the genomic location (chromosomal or plasmid) of the MDEs and their distribution pattern in all the isolates.

A strong positive correlation (Pearson coefficient =1) was observed for the co-existence of *norA, norB* and *tet38* efflux genes. This was followed by *qacA/B* and *smr* (Pearson coefficient = 0.89) which were present in 41.24% (40/97) and 44.33% (44/97) of the isolates, respectively (Table **S4**). Weak negative correlations were also observed for the co-existence of *tet*38 and other MDE genes including *nor*A, *nor*B and *qac*A/B (Table **S4**). Heatmap matrix Fig. (**1**) revealed the tendency of *nor*C, *sdr*M, *smr* and *qacA/B* to be co-absent in some *S. aureus* strains especially in the MSSA (Table **S5**).

The observed individual prevalence of the other MDEs evaluated included *nor*A (98.9%; 96/97); *nor*B (98.9%; 96/97); *mep*A (97.9%; 95/97); *tet*38 (98.9%; 96/97); *sep*A (96.9%; 94/97); *Mde*A (95.9%; 93/97); *lmrs* (88.7%; 86/97); *sdrM* (85.6%; 83/97) and *nor*C (79.4%; 77/97) (Table **S5**).

### Cluster-Dependent Heterogenic Growth Response to Methicillin and Co-Clustering of Clinical MRSA and MSSA Isolates in Relation to Their Growth Dynamics in the Presence of Methicillin

**Fig. (2):**
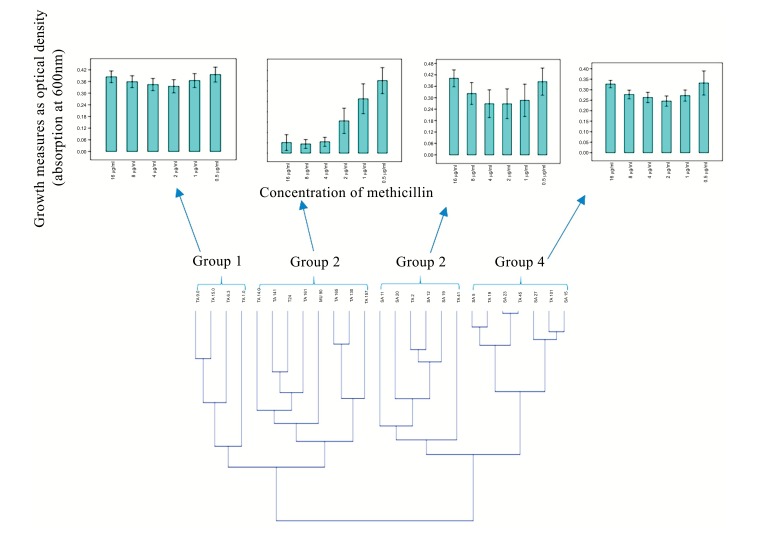
A: A dendrogram showing the co-clustering of clinical MRSA and MSSA isolates. B: Cluster-dependent heterogenic growth response to methicillin based on the general trend of the contribution of each isolates in a cluster. The error bar shows standard error from the mean. Groups 1 and 2 show exclusive clustering of MRSA while Groups 3 and 4 show co-clustering of MSSA and MRSA isolates. Samples ID with SA are MSSA and those with TA are MRSA.

**Fig. (3):**
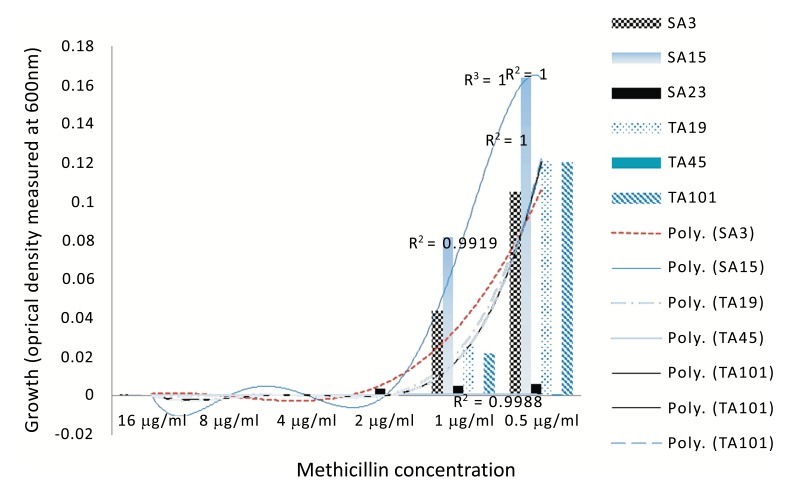
Homogenous response pattern to methicillin by MRSA and MSSA. The lines linking each methicillin concentration indicate polynomial trends of the bacterial growth. Isolates IDs with TA indicate MRSA while SA indicate MSSA.

Hierarchical clustering based on the spectrophotometric measurement of the growth of clinical *S. aureus* isolates in the presence of different concentration of methicillin, revealed two major clusters made up of four groups which showed heterogeneity in their response to methicillin Fig. (**2A**). Moreover, MRSA and MSSA isolates co-clustered (Fig. **2B**); Groups 3 and 4) in relation to their growth trend. Assessment of the individual MRSA and MSSA isolates within the co-clusters showed that they had similar growth-response phenotype (Fig. **3**). However, MRSA isolates exclusively made up other clusters (Fig. **2B**); Groups 1 and 2). There was no correlation between the specimen type or the hospital unit where the specimen were collected (Table **S6**).

The phenotypic characteristics of the four groups are as follows: Group 1: Those that were resistant at all tested concentrations of methicillin and therefore showed no dose-dependent difference in the level of growth. Group 2: Those that showed typical dose-dependent growth reduction in the presence of methicillin. Group 3: Those that appeared to display a growth trend that was directly proportional to the methicillin dose increment from 1-16 µg/ml. The *S. aureus* MU50 ATCC 700699 was clustered in this group. Group 4: Isolates that showed similar growth trend were observed in Group 3, however, with a lower average growth.

Correlation analysis of quantitative spectrophotometric growth measurements and the distribution of MDEs in the 24 *S. aureus* isolates revealed no specific correlation between the distribution pattern of MDEs and the concentrations of methicillin tested in this study (Data not shown).

### Evaluation of Visual SCs.

Visual SC determination of the 24 representative *S. aureus* isolates (based on the MDE distribution pattern) showed that two (11.8%; 2/17) MRSA and two (28.6%; 2/7) MSSA isolates had an MIC of 0.5 µg/ml; two (11.8%; 2/17) MRSA and MSSA isolates had MIC of 2 µg/ml respectively, while two (28.6%; 2/7) MSSA isolates had an MIC of 4 µg/ml Table (**2**). As expected, most of the MRSA (76.5%; 13/17) isolates were resistant to methicillin while the MSSA isolates (57.1%; 4/6) were susceptible. Intermediate resistance (1-4 spots of bacterial growth in the micro-wells) ranging from 8-16 µg/ml was observed in two (11.8%; 2/17) of the representative MRSA isolates. Intermediate resistance ranging from 1-16µg/ml was observed in four (50%; 4/8) of the MSSA (Table **S4**).

## DISCUSSION

*Staphylococcus aureus*-associated diseases remain a threat to human and animal health. Methicillin-resistant *Staphylococcus aureus* (MRSA) across the world often leave clinicians with little or no choice of treatment options [9, 10]. As the genetic diverity of this pathogen increases [11, 12] and fewer new antibiotics are entering into clinical use, efforts are being renewed to boost the activity of old antimicrobials to which pathogens have developed resistance. These efforts include the implementation of antibiotic stewardship which includes strategic use of antibiotics [13] and the development of antibiotic adjuvants such as efflux pumps inhibitors to counter resistance and/or enhance the activity of antibiotics in clinical use [14].

To contribute to this effort, this study revealed three major clusters of MDE distribution patterns in the clinical *S. aureus* isolates obtained from a hospital in South Africa suggesting that all members of the same bacterial species may not contain the same MDEs as previously suggested [15].

The prevalence of *qac*A/B gene in this study (35.08%) is higher compared to that reported in a Japanese hospital (23.6%) (16). Moreover, *smr* gene which has a lower activity compared to *qac*A/B gene (2) was present in 43.3% of the isolates and higher in prevalence compared to that of the Japanese study (4.2%) [16].

It has been shown that most efflux pumps inhibitors have been focused on *nor*A [14]. The observations in this study indicate the importance of expanding this effort to cover other MDEs. Moreover, the detection of *nor*A, *nor*B *nor*C, *mep*A and *sdr*M in all the isolates corroborates with the focus on these important MDE efflux pumps and suggest the potential for resistance to flouroquinolones incuding norfloxacin, ciprofloxacin and sparfloxacin [2, 14]. Our laboratory is looking for the possible correlation between the presence of these genes and resistance to flouroquinolones.

The high prevalence of *mde*A, *sep*A, and *mep*A is particularly worrisome and suggests a high risk of resistance to a wide range of commonly used antiseptics, biocides and antibiotics of different chemical class [2]. Similarly, *tet*38 which is involved in the extrusion of tetracycline [2] was absent only in one MRSA isolate indicating a high prevalence of this gene in the *S. aureus* isolated from patients attending the health care facility.

Interestingly, we found an isolate (TA162) which harboured only *qac*A/B and *smr* (Table S3). Repeated PCR assays indicated a consistent result for this isolate. It is not known whether this was due to mutations in the genes that prevented primer recognition of target sites in this isolate or this isolate simply evolved differently from the other isolates tested in this study. Further assessment of this isolate is the next step to be followed in our laboratory. Moreover, this study showed the co-existence and co-absence of some MDEs in the *S. aureus* strains suggesting a possible co-evolution of these genes in the pathogens. It is not certain if the co-absence of *nor*C, *sdr*M, *smr* and *qacA/B* in the MSSA strains plays a role in susceptibility to antibiotics compared to the MRSA.

Although it is not known what factor drives the different distribution of efflux genes in the *S. aureus* isolates in this study, it is thought that antibiotic resistance is a natural ecological phenomenon resulting from years of evolutionary process that may include a combination of different mechanisms [17].

It has been suggested that MDEs may also play a role in virulence of bacteria as these factors assist in colonization of the host tissue by removing host bile salts and antimicrobial peptides that may prevent colonization process [18-20]. The finding of different distribution patterns in individual isolates begs the question of ‘what roles do the different distribution patterns have in these isolates? Pearsons correlation analysis suggested that none of the combinations of MDEs evaluated in this study had an obvious effect on the β-lactam, methicilin. However, the effect of individual MDE on the resistance pattern could not be assessed using this experimental design. No correlation was observed between the MDEs distribution pattern in an isolate and SCC*mec* type (Data not shown).

By evaluating the visual MIC, SC and the growth dynamics in the presence of methicilin, the resistance phenotype of the clinical MRSA and MSSA isolates could be determined. Importantly, some MRSA and MSSA isolates demonstrated similar susceptibility even at 0.5 µg/ml methicilin, or resistance at concentration as high as 16 µg/ml (Fig. 2). This suggest that despite the lack of *mec*A genes in MSSA isolates, phenotypic resistance characteristics should not be overlooked in clinical settings.

Similarly, phenotypic assessment of the resistance mechanism in the clinical *S. aureus* isolates using spectrophotometric measurements of the growth in TSB media, showed two major clusters consisting of four groups of specific growth-response pattern showing the resistance to the model β lactam antibiotic, methicillin. The clustering of the well characterized *S. aureus* MU50 ATCC 799600 to Group 2 which showed a dose-dependent response in contrast to other groups (Fig. 2A) clearly showed that there exist phenotypic differences in the resistance pattern of clinial MRSA to methicilin and perhaps other β lactams with similar physicochemical properties. This quantitative analysis proved valuable in being able to reveal the growth dynamics of the isolates evaluated and showed that a group of isolates (Fig. **2B**); Groups 3 and 4) actually had higher growth as the methicillin concentration increased. Another instance of the advantage provided by spectrophotometric bacterial growth measurement can be seen in Group 3 (Fig. **2A**) in which isolates SA11, SA28, TA2, SA12, SA19 and TA41 showed increasing optical densities in response to higher concentrations (4-16 µg/ml) of methicillin, despite the fact that the MSSA isolates SA11, SA28 and SA12 were classified as susceptible at this concentrations (Table 2) based on the visual MIC assessment criteria for describing susceptibility as indicated in the method section. Interestingly, these isolates co-clustered with MRSA isolates TA41 and TA12 which displayed resistance at these concentrations thus indicating that the co-clustering was based mainly on the growth dynamics and independent of the genotype. These observations are contrary to reported hormesis effect where low doses of antibiotics are associated with increased bacterial growth [21, 22].

Subsistence of environmental bacteria on antibiotics has been previously reported [23]. However, to our knowledge, this event has not been reported in clinical *S. aureus* isolates. Although it is not known if this observation was a result of direct utilization of methicillin as a carbon source or a trigger of other mechanisms that promote the bacterial growth, this observation represents a potential *S. aureus* resistance mechanism to methicilin and perhaps other β lactam antibiotics with similar structural and physicochemical properties. Theoretically, the clinical importance of these findings is that patients infected with these set of MSSA isolates may likely be prescribed/administered with antibiotic regimen that may not be able to kill the pathogen.

In conclusion, this study describes the diversity of MDEs in a hospital in South Africa and shows that there were at least three major distribution patterns of MDEs in the clinical isolates evaluated. This information is important for the development of antibiotic adjuvants that target the inhibition of MDE in *S. aureus*. In addition, we report that *S. aureus* isolates, be it of MRSA or MSSA genotype, do not contain the same set of MDEs despite being recovered from the same hospital setting. The high prevalence of MDEs suggestive of resistance to a broad spectrum of biocides and fluoroquinolones is particularly disturbing. A limitation of this study is the lack of CFU/mL data which would have shown the actual log increase or decrease in bacterial growth. However, a previous report demonstrated a strong correlation between optical density (absorption) measurement of *S. aureus* growth and CFU/mL [24-30]. Finally, at least four specific growth-response patterns of *S. aureus* in the presence of a model β lactam antibiotic (methicillin) were observed and included the enhancement of *S. aureus* growth in the presence of the antimicrobial agent.

## SUPPLEMENTARY MATERIAL

Supplementary material is available on the publishers Website along with the published article.

## Figures and Tables

**Fig. (1):**
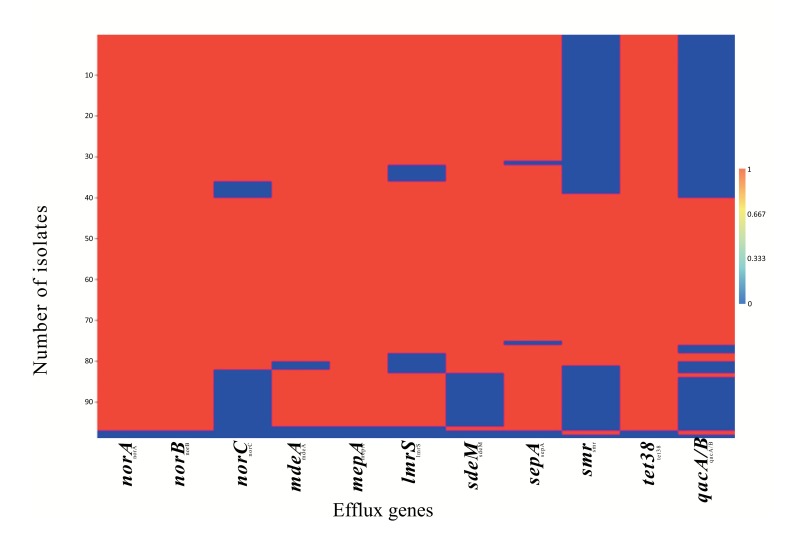
Heatmap matrix showing a visual representation of the distribution pattern of the 11 MDEs in MRSA and MSSA isolates. The blue reagions indicate the set of isolates in which an MDE was not detected while the red regions indicate the set of isolates in which the MDEs were detected by PCR. A similarity in the distribution pattern of *qac*A/B and *smr*; *nor*A and *nor*B can be seen.

**Table 1:** List of primers for multiplex PCR assays.

**Primer code/target gene**	Sequence (5' to 3')*	**Amplicon size**	**Reference**
qacA/B (F)	CTATGGCAATAGGAGATATGGTGT	321 bp	(24)
qacA/B (R)	CCACTACAGATTCTTCAGCTACATG		
qacC (F)	AACAATGCAACACCTACCACT	157 bp	(24)
qacC (F)	AACGAAACTACGCCGACTATG		
smr (F)	CTATGGCAATAGGAGATATGGTGT	417 bp	(25)
smr (R)	CCACTACAGATTCTTCAGCTACATG		
norA (F)	TTCACCAAGCCATCAAAAAG	95 bp	(26)
norA (R)	CCATAAATCCACCAATCCC		
norB (F)	AGCGCGTTGTCTATCTTTCC	213 bp	(27)
norB (R)	GCAGGTGGTCTTGCTGATAA		
norC (F)	AATGGGTTCTAAGCGACCAA	216 bp	(27)
norC (R)	ATACCTGAAGCAACGCCAAC		
mepA (F)	TGCTGCTGCTCTGTTCTTTA	198 bp	(27)
mepA (R)	GCGAAGTTTCCATAATGTGC		
mdeA (F)	GTTTATGCGATTCGAATGGTTGGT	155 bp	(28)
mdeA (B)	AATTAATGCAGCTGTTCCGATAGA		
sepA (F)	GCAGTCGAGCATTTAATGGA	103 bp	(27)
sepA (R)	ACGTTGTTGCAACTGTGTAAGA		
lmrS (F)	TGCAGTTAAATGCGATGGCG	135 bp	This study
lmrS (R)	GAAATCTCACATGGCACGGC		
sdrM (F)	GGCAATGATCGCAATCGGTA	126 bp	This study
sdrM (R)	ATGGGCATAGTTGGCAGTGT		
tet38 (F)	AGTTGGCAAGCGACATTAGC	212 bp	This study
tet38 (R)	GTCTCTGCAGCAGCTAAACC		
MecA1-F	GTAGAAATGACTGAACGTCCGATAA	310 bp	(8)
MecA2-R	CCAATTCCACATTGTTTCGGTCTAA		
314F (Eubacteria 16S rDNA V3 region)	CCTACGGGAGGCAGCAG	200 bp	(29)
518R (Eubacteria 16S rDNA V3 region)	ATTACCGCGGCTGCTGG		

**Table 2:** Visual MIC and SC determination on the 24 representative S. aureus isolates (based on the MDE distribution pattern).

**Methicillin concentration**						
**Isolates ID†**	16 µg/ml	8 µg/ml	4 µg/ml	2 µg/ml	1 µg/ml	0.5 µg/ml
**TA45**	S	S	S	S	S	S
**TA63**	R	R	R	R	R	R
**SA27**	IR	IR	IR	IR	R	R
**SA28**	S	S	S	IR	R	R
**TA2**	IR	IR	R	R	R	R
**TA101**	IR	IR	R	R	R	R
**SA12**	S	S	S	S	R	R
**SA15**	S	S	S	S	R	R
**TA165**	S	S	S	S	IR	R
**TA168**	R	R	R	R	R	R
**SA11**	S	S	S	R	R	R
**SA19**	IR	IR	R	R	R	R
**TA41**	IR	IR	R	R	R	R
**TA57**	R	R	R	R	R	R
**TA162**	S	S	S	S	S	S
**TA98**	R	R	R	R	R	R
**TA130**	R	R	R	R	R	R
**SA3**	S	S	S	S	IR	R
**TA141**	R	R	R	R	R	R
**SA23**	S	S	S	S	S	S
**TA19**	S	S	S	S	IR	R
**TA70**	R	R	R	R	R	R
**TA161**	R	R	R	R	R	R
**TA24**	R	R	R	R	R	R

Sample IDs with ‘SA’ are MSSA while those with ‘TA’ are MRSA isolates
