# Supplementary material for: Diversity of Multidrug Efflux Genes and Phenotypic Evaluation of the In vitro Resistance Dynamics of Clinical Staphylococcus Aureus Isolates Using Methicillin; a Model β-lactam
Source: Open Microbiol J. 2017 Jun 30;11:132–41. doi: 10.2174/1874285801711010132 (PMC5543611; doi:10.2174/1874285801711010132)
Supplement: Supplementary file 1 [file TOMICROJ-11-132_SD1.pdf]

Send Orders for Reprints to reprints@benthamscience.ae

BENTHAM OPEN

CrossMark

The Open Microbiology Journal

Supplementary Material

Content list available at: www.benthamopen.com/TOMICROJ/

DOI: 10.2174/1874285801711010132

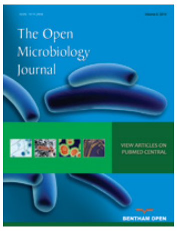

Diversity of Multidrug Efflux Genes and Phenotypic valuation of the *In vitro* Resistance Dynamics of Clinical *Staphylococcus Aureus* Isolates Using Methicillin; a Model β-lactam

John Francis Antiabong*, Marleen M. Kock, Nontombi M. Mbellea and Marthie M. Ehlers

University of Pretoria Medical Microbiology South Africa

SUPPLEMENTARY MATERIAL

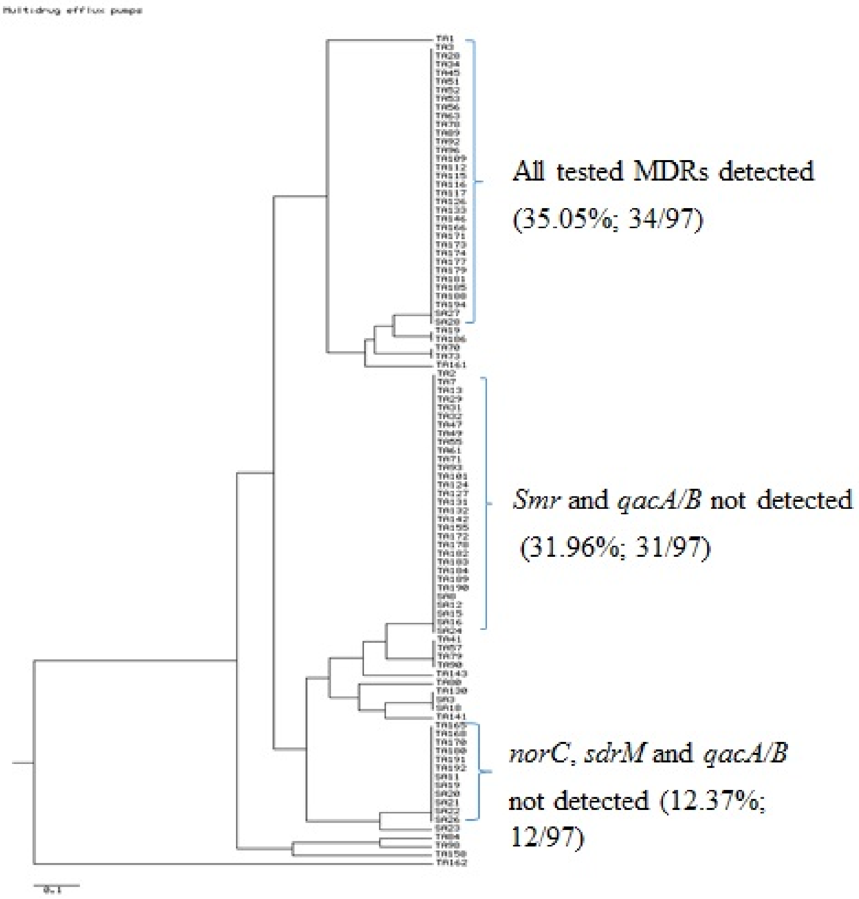

Supp. (1). Hierarchical clustering showing three major clusters of the 11 MDEs in MRSA and MSSA isolate. Other minor clusters/outliers contained 1 to 4 isolates and made up 21.2% of the total isolates investigated. Samples IDs with SA are MSSA and those with TA are MRSA isolates.

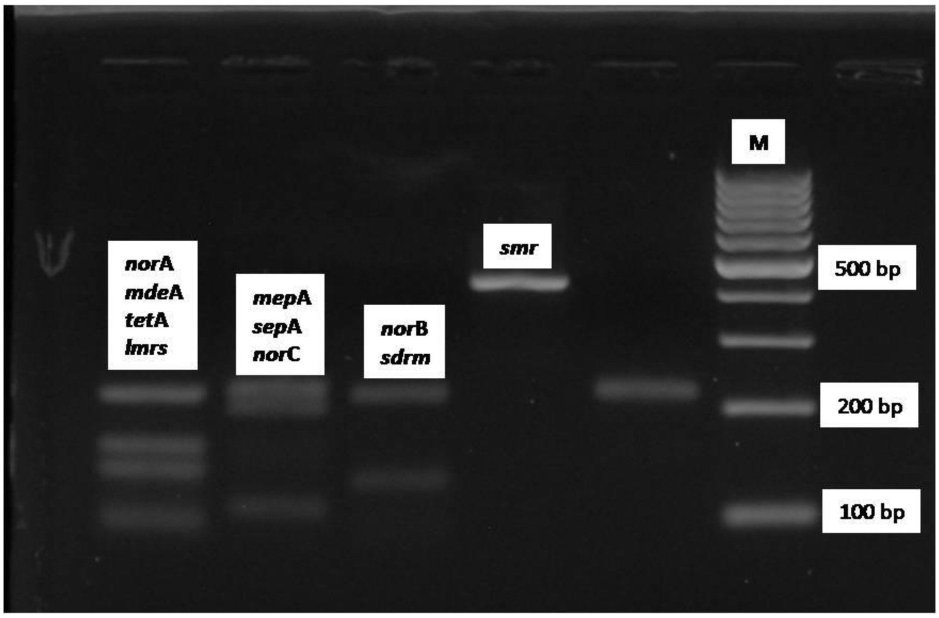

Supp. (2). Agorose electrophoretic gel pattern of the multidrug efflux genes (MDEs) using the mutiplex PCR that is based on the previously described cycliplex PCR strategy described by Antiabong and colleagues (6). All the targets were amplified in diffeent tubes using one cycling PCR condition at the same time thereby reducing time and ensuring accuracy. The gel shows the amplicon bands of norA (95 bp), mdeA (155 bp), tetA (212 BP), lmrs (135 bp) in the first lane, the second lane includes mepA (198 bp), sepA (103 bp), norC (216 bp) while the third lane comprises of norB (213 bp) and sdmr (126 bp). The forth and fifth lanes are smr (417 bp) and qacA/B (321 bp). M is 100 bp Generuler (Thermo Scientific, South Africa). The pattern shown is based on gDNA extract from Staphylococcus aureus MU50 ATCC 700699.

Table S1. Multi-drug efflux pumps described for *Staphylococcus aureus.*

Efflux Pump	Family[a]	Regulator(s)[b]	Substrate Specificity
NorA	MFS	MgrA, NorG(?)	Hydrophilic fluoroquinolones (ciprofloxacin, norfloxacin) QACs (tetraphenylphosphonium, benzalkonium chloride) Dyes (e.g. ethidium bromide, rhodamine)
NorB	MFS	MgrA, NorG	Fluoroquinolones (e.g. hydrophilic: ciprofloxacin, norfloxacin and hydrophobic: moxifloxacin, sparfloxacin) Tetracycline QACs (e.g. tetraphenylphosphonium, cetrimide) Dyes (e.g. ethidium bromide)
NorC	MFS	MgrA(?), NorG	Fluoroquinolones (e.g. hydrophilic: ciprofloxacin and hydrophobic: moxifloxacin) Dyes (e.g. rhodamine)
MepA	MATE	MepR	Fluoroquinolones (e.g. hydrophilic: ciprofloxacin, norfloxacin and hydrophobic: moxifloxacin, sparfloxacin) Glycylcyclines (e.g. tigecycline) QACs (e.g. tetraphenylphosphonium, cetrimide, benzalkonium chloride) Dyes (e.g. ethidium bromide)
MdeA	MFS	n.i.	Hydrophilic fluoroquinolones (e.g. ciprofloxacin, norfloxacin) Virginiamycin, novobiocin, mupirocin, fusidic acid QACs (e.g. tetraphenylphosphonium, benzalkonium chloride, dequalinium) Dyes (e.g. ethidium bromide)
SepA	n.d.	n.i.	QACs (e.g. benzalkonium chloride) Biguanidines (e.g. chlorhexidine) Dyes (e.g. acriflavine)
SdrM	MFS	n.i.	Hydrophilic fluoroquinolones (e.g. norfloxacin) Dyes (e.g. ethidium bromide, acriflavine)
LmrS	MFS	n.i.	Oxazolidinone (linezolid) Phenicols (e.g. choramphenicol, florfenicol) Trimethoprim, erythromycin, kanamycin, fusidic acid QACs (e.g. tetraphenylphosphonium) Detergents (e.g. sodium dodecyl sulphate) Dyes (e.g. ethidium bromide)
QacA	MFS	QacR	QACs (e.g. tetraphenylphosphonium, benzalkonium chloride, dequalinium) Biguanidines (e.g. chlorhexidine) Diamidines (e.g. pentamidine) Dyes (e.g. ethidium bromide, rhodamine, acriflavine)
QacB	MFS	QacR	QACs (e.g. tetraphenylphosphonium, benzalkonium chloride) Dyes (e.g. ethidium bromide, rhodamine, acriflavine)
Smr	SMR	n.i	QACs (e.g. benzalkonium chloride, cetrimide) Dyes (e.g. ethidium bromide)
QacG	SMR	n.i	QACs (e.g. benzalkonium chloride, cetyltrymethylammonium) Dyes (e.g. ethidium bromide)
QacH	SMR	n.i	QACs (e.g. benzalkonium chloride, cetyltrymethylammonium) Dyes (e.g. ethidium bromide)
QacJ	SMR	n.i	QACs (e.g. benzalkonium chloride, cetyltrymethylammonium) Dyes (e.g. ethidium bromide)

Adapted from Costa *et al.* (2)

[a]n.d: The family of transporters to which *Sep*A belongs is not elucidated to date.

[b]n.i: The transporter has no regulator identified to date. QACs: quaternary ammonium compounds

Table S2. PCR conditions and banding pattern/M-PCR target combinations.

Mutidrug efflux genes	№ of Isolates (percentage)	Isolates in the group
lmrs mdeA mepA norA norB norC qacA sdrM sepA smr tet38	34*	SA27 TA188 TA133 TA174 TA34 TA146 TA126 TA116 TA171 TA52 SA28 TA1 TA89 TA53 TA78 TA45 TA117 TA177 TA194 TA51 TA173 TA96 TA185 TA179 TA28 TA112 TA3 TA181 TA166 TA63 TA115 TA56 TA92 TA109
lmrs mdeA mepA norA norB norC sdrM sepA smr tet38	2	TA19 TA186
lmrs mdeA mepA norA norB norC qacA sdrM smr tet38	1	TA161
mdeA mepA norA norB norC qacA sdrM sepA smr tet38	2	TA70 TA73
lmrs mdeA mepA norA norB norC sdrM sepA tet38	31**	TA71 TA189 SA8 TA132 SA16 TA13 TA182 TA31 TA184 TA101 SA24 TA190 TA142 SA15 TA2 TA183 TA49 TA131 TA124 SA12 TA127 TA178 TA47 TA32 TA7 TA29 TA172 TA93 TA155 TA61 TA55
lmrs mdeA mepA norA norB sdrM sepA smr tet38	1	TA141
lmrs mdeA mepA norA norB norC sdrM tet38	1	TA143
mdeA mepA norA norB norC sdrM sepA tet38	4	TA41 TA57 TA90 TA79
mepA norA norB norC sdrM sepA smr tet38	1	TA84
lmrs mdeA mepA norA norB sdrM sepA tet38	3	TA130 SA3 SA18
lmrs mdeA mepA norA norB qacA sepA tet38	1	SA23
mepA norA norB norC sdrM sepA tet38	1	TA98
lmrs mdeA mepA norA norB sepA tet38	12***	SA20 SA11 TA192 TA191 SA26 TA170 SA19 TA168 TA165 SA21 SA22 TA180
mdeA mepA norA norB sdrM sepA tet38	1	TA80
norA norB sdrM sepA tet38	1	TA158
qacA smr	1	TA162

*All tested MDEs detected (35.05%)
***Smr* and *qacA/B* not detected (31.96%)
****norC*, *sdrM* and *qacA/B* not detected (12.37%)

Table S3. Hospital units and specimen sources associated with each cluster of clinical *S. aureus* isolates displaying heterogenic growth pattern.

GROUP 1			GROUP 2			GROUP 3			GROUP 4		
**Isolate ID**	**Hospital Unit**	**Specimen type**	**Isolate ID**	**Hospital Unit**	**Specimen type**	**Isolate ID**	**Hospital Unit**	**Specimen type**	**Isolate ID**	**Hospital Unit**	**Specimen type**
**TA70**	Orthopaediatric	Pus swab	**T158**	Surgery	Pus swab	**SA11**	Cystic fibrosis	Sputum	**SA3**	Cystic fibrosis	Sputum
**TA63**	High care	Central Venous Pressure tip	**T141**	Surgery and Trauma	Blood culture	**SA28**	Cystic fibrosis	Sputum	**TA19**	Orthopaediatric	Tissue
**TA98**	Cardiothoracic surgery	Endotracheal aspirate	**T24**	Orthopaediatric	Pus swab	**TA2**	Urology	Blood culture	**SA23**	Cystic fibrosis	Sputum
**TA158**	High care	Central Venous Pressure tip	**T161**	Medical pulmonology	Endotracheal aspirate	**SA12**	Cystic fibrosis	Sputum	**TA45**	Baby room ICU	Blood culture
-	-	-	**MU50**	N/A	N/A	**SA19**	Cystic fibrosis	Sputum	**SA27**	Cystic fibrosis	Sputum
-	-	-	**T165**	Medical pulmonology	Sputum	**TA43**	Short stay	Blood culture	**TA101**	High care	Pus swab
-	-	-	**T130**	Surgery and Trauma	Endotracheal aspirate	-	-	-	**SA15**	Cystic fibrosis	Sputum
-	-	-	**T57**	Orthopaediatric	Tissue	-	-	-	-	-	-

MU50: *Staphylococcus aureus* MU50 ATCC 700699 N/A: not applicable

Table S4. Pearson's correlation analysis showing the probability the co-existence of multidrug efflux genes in 97 clinical *S. aureus* strains.

**Pearson's correlation**											
	**norA**	**norB**	**norC**	**mdeA**	**mepA**	**lmrS**	**sdrM**	**sepA**	**smr**	**tet38**	**qacA/B**
**norA**		0	0.005556	5.98E-12	1.93E-24	7.72E-05	0.00058	7.73E-16	0.85177	0	0.78263
**norB**	1		0.005556	5.98E-12	1.93E-24	7.72E-05	0.00058	7.73E-16	0.85177	0	0.78263
**norC**	0.27674	0.27674		0.02955	0.000559	0.2779	1.17E-24	0.15359	0.000312	0.005556	0.000972
**mdeA**	0.6226	0.6226	0.21882		2.33E-20	7.02E-12	0.11403	1.42E-05	0.87525	5.98E-12	0.34504
**mepA**	0.81228	0.81228	0.34069	0.76649		6.38E-07	0.011188	1.40E-09	0.72334	1.93E-24	0.80238
**lmrS**	0.38663	0.38663	0.11012	0.621	0.47599		0.87747	0.01761	0.45654	7.72E-05	0.25047
**sdrM**	0.3398	0.3398	0.81441	0.15983	0.25399	0.015694		0.047807	0.001582	0.00058	0.020223
**sepA**	0.69978	0.69978	0.1445	0.42119	0.56224	0.23815	0.19944		0.78943	7.73E-16	0.69333
**smr**	-0.01902	-0.01902	0.35498	0.01598	0.036024	0.075684	0.31346	-0.02718		0.85177	2.21E-36
**tet38**	1	1	0.27674	0.6226	0.81228	0.38663	0.3398	0.69978	-0.01902		0.78263
**qacA/B**	-0.02808	-0.02808	0.32653	0.0959	0.025472	0.11659	0.23312	-0.04013	0.89812	-0.02808	

Table S5. Hospital units and specimen sources associated with each cluster of clinical S. aureus isolates displaying heterogenic growth pattern.

**GROUP 1**			**GROUP 2**			**GROUP 3**			**GROUP 4**		
**Isolate ID**	**Hospital Unit**	**Specimen type**	**Isolate ID**	**Hospital Unit**	**Specimen type**	**Isolate ID**	**Hospital Unit**	**Specimen type**	**Isolate ID**	**Hospital Unit**	**Specimen type**
**TA70**	Orthopaediatric	Pus swab	**T158**	Surgery	Pus swab	**SA11**	Cystic fibrosis	Sputum	**SA3**	Cystic fibrosis	Sputum
**TA63**	High care	Central Venous Pressure tip	**T141**	Surgery and Trauma	Blood culture	**SA28**	Cystic fibrosis	Sputum	**TA19**	Orthopaediatric	Tissue
**TA98**	Cardiothoracic surgery	Endotracheal aspirate	**T24**	Orthopaediatric	Pus swab	**TA2**	Urology	Blood culture	**SA23**	Cystic fibrosis	Sputum
**TA158**	High care	Central Venous Pressure tip	**T161**	Medical pulmonology	Endotracheal aspirate	**SA12**	Cystic fibrosis	Sputum	**TA45**	Baby room ICU	Blood culture
-	-	-	**MU50**	N/A	N/A	**SA19**	Cystic fibrosis	Sputum	**SA27**	Cystic fibrosis	Sputum
-	-	-	**T165**	Medical pulmonology	Sputum	**TA43**	Short stay	Blood culture	**TA101**	High care	Pus swab
-	-	-	**T130**	Surgery and Trauma	Endotracheal aspirate	-	-	-	**SA15**	Cystic fibrosis	Sputum
-	-	-	**T57**	Orthopaediatric	Tissue	-	-	-	-	-	-

MU50: Staphylococcus aureus MU50 ATCC 700699
N/A: not applicable

© 2017 Antiabong *et al.*

This is an open access article distributed under the terms of the Creative Commons Attribution 4.0 International Public License (CC-BY 4.0), a copy of which is available at: https://creativecommons.org/licenses/by/4.0/legalcode. This license permits unrestricted use, distribution, and reproduction in any medium, provided the original author and source are credited.
